# Molecular pathways and targeted therapies in head and neck cancers pathogenesis

**DOI:** 10.3389/fonc.2024.1373821

**Published:** 2024-06-17

**Authors:** Marian Constantin, Mariana Carmen Chifiriuc, Coralia Bleotu, Corneliu Ovidiu Vrancianu, Roxana-Elena Cristian, Serban Vifor Bertesteanu, Raluca Grigore, Gloria Bertesteanu

**Affiliations:** ^1^ Department of Microbiology, Institute of Biology of Romanian Academy, Bucharest, Romania; ^2^ The Research Institute of the University of Bucharest, ICUB, Bucharest, Romania; ^3^ Microbiology Immunology Department, Faculty of Biology, University of Bucharest, Bucharest, Romania; ^4^ Romanian Academy, Bucharest, Romania; ^5^ Cellular and Molecular Pathology Department, Ştefan S. Nicolau Institute of Virology, Bucharest, Romania; ^6^ DANUBIUS Department, National Institute of Research and Development for Biological Sciences, Bucharest, Romania; ^7^ Department of Biochemistry and Molecular Biology, Faculty of Biology, University of Bucharest, Bucharest, Romania; ^8^ ENT, Head& Neck Surgery Department, Carol Davila University of Medicine and Pharmacy, Coltea Clinical Hospital, Bucharest, Romania

**Keywords:** head and neck cancer, molecular pathways, targeted therapy, monoclonal antibodies, genomic, epigenetic, ncRNA

## Abstract

The substantial heterogeneity exhibited by head and neck cancer (HNC), encompassing diverse cellular origins, anatomical locations, and etiological contributors, combined with the prevalent late-stage diagnosis, poses significant challenges for clinical management. Genomic sequencing endeavors have revealed extensive alterations in key signaling pathways that regulate cellular proliferation and survival. Initiatives to engineer therapies targeting these dysregulated pathways are underway, with several candidate molecules progressing to clinical evaluation phases, including FDA approval for agents like the EGFR-targeting monoclonal antibody cetuximab for K-RAS wild-type, EGFR-mutant HNSCC treatment. Non-coding RNAs (ncRNAs), owing to their enhanced stability in biological fluids and their important roles in intracellular and intercellular signaling within HNC contexts, are now recognized as potent biomarkers for disease management, catalyzing further refined diagnostic and therapeutic strategies, edging closer to the personalized medicine desideratum. Enhanced comprehension of the genomic and immunological landscapes characteristic of HNC is anticipated to facilitate a more rigorous assessment of targeted therapies benefits and limitations, optimize their clinical deployment, and foster innovative advancements in treatment approaches. This review presents an update on the molecular mechanisms and mutational spectrum of HNC driving the oncogenesis of head and neck malignancies and explores their implications for advancing diagnostic methodologies and precision therapeutics.

## 1 Introduction

From the very heterogeneous group of head and neck cancers (HNC) (1), the head and neck squamous cell carcinomas (HNSCC) produced by the transformation of the squamous cell epithelia lining the oral cavity, pharynx and larynx are the sixth most common type of cancer (2–4), with 700,000 - 900,000 new cases recorded annually (2, 4), responsible for 0.5% (in case of oropharynx cancers) to 1.9% (for lip and oral cavity cancers) of deaths from all cancers combined (2).

The most invoked etiological factors are smoking, alcohol consumption (5, 6) and HPV (human papillomavirus) infections (7a; 7b; 8–14). In addition, several factors contribute more or less to HNC development, including EBV (Epstein-Barr virus) infections (15, 16), laryngopharyngeal reflux (17), chewing betel quid (Areca nuts) (18, 19), poor oral hygiene (20), oral dysbiosis (21), pro-inflammatory diet (22, 23), and inhalation of airborne pollutants (24, 25). With the development of molecular diagnostic techniques, many genetic aberrations (4, 26–28) and epigenetic factors (29) have been revealed.

HNC presents significant challenges in terms of both diagnosis and treatment. Late diagnosis due to the lack of effective screening strategies often complicates treatment options and reduces overall survival rates (30). The heterogeneous nature of HNC further adds to the complexity, requiring tailored approaches for different subtypes (24, 31–34). Moreover, the aggressive nature of traditional treatments like chemoradiotherapy can lead to significant side effects and reduced quality of life for patients (35).

The choice of the appropriate therapeutic strategy must consider the anatomical site, tumor stage, and etiological factors and could include surgery, radiotherapy, chemotherapy, immunotherapy, and, more recently, targeted therapies. Despite the poor outcomes in the advanced stages (36), the latter is expected to appropriately address the high heterogeneity of HNC. Consequently, it will contribute to better patient outcomes and improved 5-year survival rates, which currently average is around 50% (2, 33, 37).

Various clinical trials have been undertaken around the world on patients with HNSCC. In the clinicaltrials.gov database, 1266 clinical studies with the condition/disease ‘HNSCC’ have been registered since 2022. Recently, Goel and collaborators summarized the results of a large number of clinical trials examining the efficacy of immunotherapy and molecular-targeted treatments. A total of 393 studies were completed out of 1266 trials registered, while 590 studies were still recruiting. Docetaxel, cisplatin, and 5-fluorouracil (5-FU) have been identified as the most often used medications in clinical trials worldwide (38), but monoclonal antibody-based medicines such as nivolumab, pembrolizumab, cetuximab, panitumumab, zalutumumab, and nimotuzumab also hold significant promise for future therapeutic applications (39–41). However, the poor prognosis for many HNC patients and high rates of locoregional recurrence and metastasis advocate for better screening methods, more effective and tolerable, personalized treatment strategies (42). This paper reviews the molecular mechanisms involved in HNC, summarizing the signaling pathways harboring genetic aberrations (RAS–RAF–MEK–ERK, PIK3–AKT–mTOR, WNT/beta-catenin, JAK-STAT, NOTCH, and HIF–VEGF), epigenetic mechanisms and the roles of ncRNAs and tumor microenvironment in neoplastic progression. We also tried to summarize the therapies targeting the abnormal functioning of these signaling pathways and their efficacy.

## 2 HNC molecular pathogenesis

**Figure 1:**
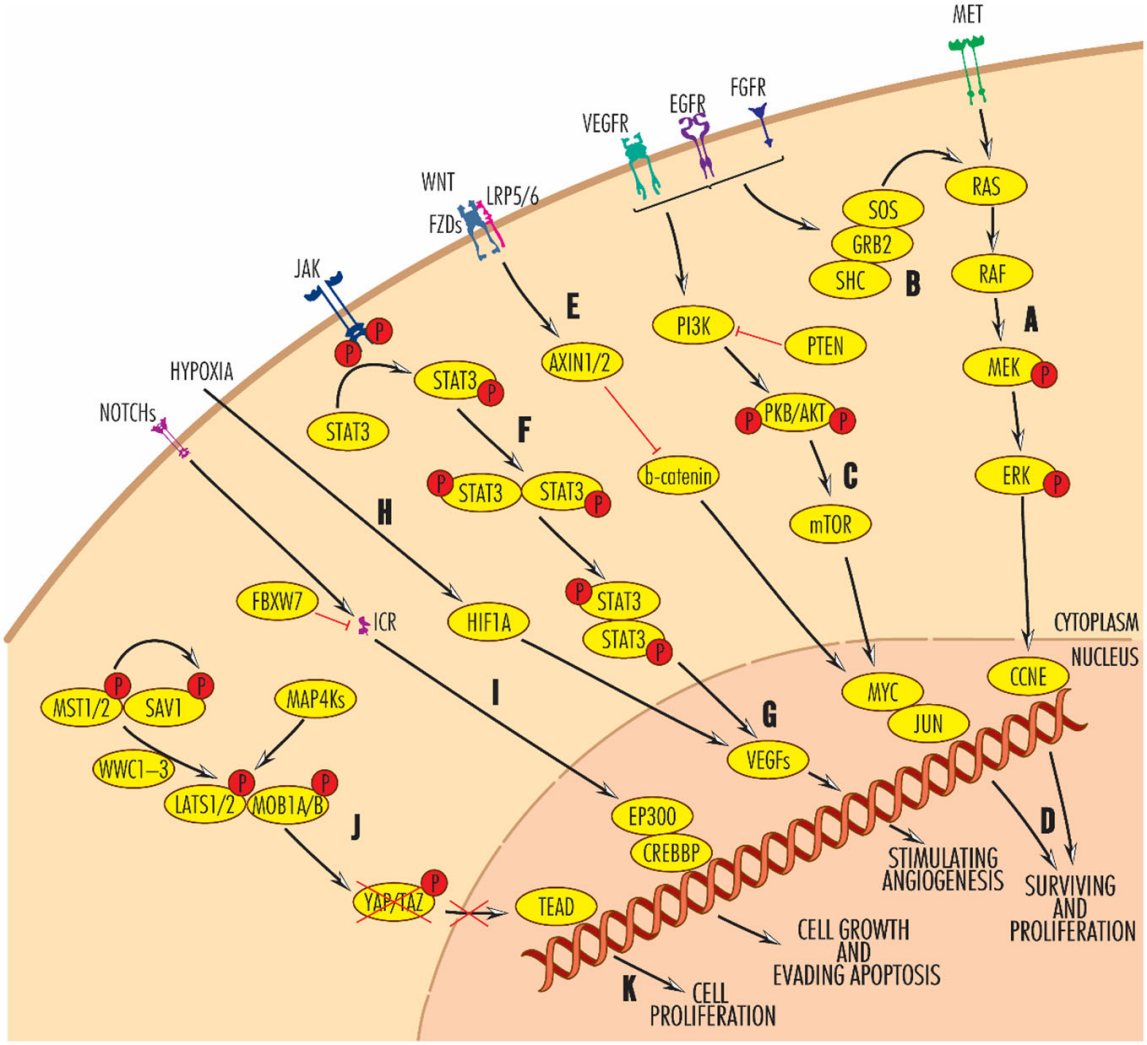
Main signaling pathways disregulated in HNC at the genomic or transcriptional level. Black arrow-ended lines indicate activation and red bar-ended lines indicate inhibition. RAS–RAF–MEK–ERK **(A, B)** and PIK3–AKT–mTOR **(C)** signaling pathways are involved in promoting cell survival and proliferation **(D)**, antagonistically to the WNT pathway **(E)**, in which AXN1/2 blocks beta-catenin activity. By enhancing JAK/STAT signaling **(F)**, VEGF proteins **(G)**, the main angiogenic molecules, are activated. Alternatively, VEGF expression is also modulated by hypoxia-induced factor 1A **(H)**. Frequently reported in HNCs, angiogenesis builds the vasculature through which the tumors are supplied with oxygen and allows their progression and a worse prognosis. On the other hand, the NOTCH signalling pathway is involved in cell growth and evasion of apoptosis **(I)**. Very important in the control of tumour processes in head and neck cancers is the Hippo pathway **(J)**, which, when activated, phosphorylates YAP/TAZ, causing its intracytoplasmic sequestration and degradation, preventing its translocation to the nucleus and activation of TEAD. TEAD activation leads to cell proliferation. For further details, please see the text.

The development of HNC is a multistep process in which mutations are accumulating in the main cellular signaling pathways that regulate proliferation, cell death, angiogenesis and immune system functions (3) (**Figure 1**).

### 2.1 Cell cycle deregulation in HNSCC

The most affected cell cycle regulating genes in HNC are *TP53*, *RB*, *CCND1*, *CDKN2A/INK4*, and *CDK6*, unblocking the G1/S transition. Thus, TP53 protein function is impaired in 50–80% of cases, the mutations in the *TP53* gene in HNSCC being the seventh most frequent in cancer diagnosis worldwide (43). Several types of mutations (deletions, insertions, and frameshift mutations, and point mutations) in the TP53 protein are associated with increased risk of progression from mild dysplasia to invasive carcinoma and unfavorable tumor progression (33, 44–47). Specifically, TP53 missense mutations in the DNA binding region are significantly enriched in metastases and are associated with a common fragile site in chromosome 11, leading to amplification and overexpression of genes with established role in metastasis (45). In HPV-positive HNC, *TP53* mutations are sporadic, as the function of this gene is selectively abrogated by viral proteins E6 and E7.

The *RB1* gene function is altered by unilateral or bilateral deletions, methylation of the RB1 promoter, or point mutations (48).

The *CCND1* gene is overexpressed in 30%-46% of cases, leading to G1 phase shortening and rapid entry into the S phase, bypassing the influence of growth factors and increasing the proliferation rate of gene-defective cells. These alterations are reported in cancers with frequent recurrence, lymph node metastasis, and reduced survival rate (49, 50).

After *CCND1*, the third most frequent alteration in HNC is the potent inhibitor *CDKN2A*, a regulator of cyclin activity and progression to S phase. *CDKN2A* alterations including deletions (more common in HPV-negative tumors), hypermethylation, and, less commonly, mutations in exon 2 (rarest in oropharyngeal tumors) are associated with metastatic cancers with poor prognosis (51). In laryngeal squamous cell carcinomas, mutations in the *CDKN2A* gene occur in approximately 14% of cases (52). *CDK6* is overexpressed in some oral tumors and correlates with tumor stage advancement and progression (36).

### 2.2 DNA repair pathway

The DNA repair pathway is a mechanism by which cells in the G1 or G2 phase are halted at checkpoints to identify and correct errors in the nucleotide sequence of nuclear genetic material. This process is initiated by the detection of DNA lesions and is activated by the ATM (ataxia-telangiectasia mutated) and ATR (ataxia-telangiectasia and Rad3 related) kinases, along with proteins such as BRCA1, BRCA2, (Mediator of DNA Damage Checkpoint 1) and T53BP1 (Tumor Protein P53 Binding Protein 1). Additionally, the synthesis of PALB2 (partner and locator of BRCA2), which interacts with BRCA1 and BRCA2, plays a crucial role in the DNA repair process.

In HNC, the most mutated genes in this signaling pathway are *ATM* (25%), *BRCA2* (9.2%), *BRCA1* (5.75%), and *ATR* (4.36%) (53–56). Recent studies have revealed that the disrupted expression of specific components within the DNA repair machinery, stemming from mutations in key pathway genes like ATM and BRCA1, can yield valuable prognostic insights for HNC patients (56, 57).

The alterations in critical cellular signaling pathways governing proliferation and cell survival have been exploited to create customized treatments for HNC individuals. The increasing interest in exploring the cell cycle (CDK4/6, CCND1, CDKN2A) and the DNA repair pathways (BRCA, ATM, ATR) is demonstrated by clinical trials (NCT03356223, NCT03065062, NCT03024489, NCT05878964, NCT04576091, NCT04491942, NCT02567422) actively investigating their potential in the broader context of HNC such as CDK4/6 inhibitors for HPV-negative tumors (42).

### 2.3 RAS–RAF–MEK–ERK signaling pathway

This pathway involves numerous proteins and receives biological signals from the extracellular space through various ligand-receptor pairs, including TGFα and EGF–EGFR/ERBB1/HER1, ERBB2/HER2, PDGF–PDGFRA and PDGFRB, IGF–IGF1R, FITL–KIT/c-KIT, FLT3L–FLT3, HGF–MET, and FGF–FGFR. These signals are transmitted into the nucleus, where they activate genes involved in cell proliferation and differentiation, inflammation, evasion of apoptosis, and support of angiogenesis (58–63). Interaction with cytokine ligands activates transmembrane receptors and recruits the growth factor receptor-bound protein 2 (GRB2) adaptor protein, which interacts with SOS1 protein (the human homolog of *Drosophila* son of sevenless 1). This is a RAS-specific guanine nucleotide exchange factor and reacts with RAS family members, the core proteins of this signaling pathway (64, 65). The RAS family of GTPases comprises three members, KRAS (Kirsten RAS oncogene homolog), HRAS (Harvey RAS oncogene homolog), and NRAS (Neuroblastoma RAS oncogene homolog), which transmit the signals downstream to RAFs (from the canonical RAS–RAF–MEK–ERK pathway), RALGDS (from the RALGDS–RAL–PLD1 signaling pathway), RASSF1 (from the RASSF1–MST1 signaling pathway) or PI3K (from the PI3K–AKT–mTOR signaling pathway) (66, 67). In the canonical RAS–RAF–MEK–ERK pathway, RAS proteins are the first members of a four-step cascade of cytoplasmic protein kinase kinases, which include: (1) RAF (rapidly accelerated fibrosarcoma kinase), RAF1/c-RAF, BRAF and ARAF family of kinases, designated MAPKKK or MAP3K; (2) MEK (mitogen-activated protein kinases); (3) ERK (extracellular signal-regulated kinase) (68, 69). In the RAS–RAF–MEK–ERK signaling pathway, overexpression of EGFR/ERBB1 occurs early in the progression of HNSCC, leading to poor prognosis (70). In tumor xenografts of HPV-positive HNSCC, it enhances the response to radiotherapy by decreasing the expression of the HPV protease E6 and affecting DNA repair mechanisms (71).

In most HNC, members of the RAS gene family are most frequently mutated, followed at a long distance by those of the RAF gene family (72). In a study of 51 patients with HNSCC (with higher prevalence in the larynx and trachea area), mutations of the *KRAS* gene (sometimes designated *KRAS1* or *KRAS2*) were detected in 35% of cases, and mutations of the *HRAS* gene in 33% of cases, with the caveat that *KRAS* mutations, *HRAS* mutations, and HPV infection are mutually exclusive (68). The most frequent mutations (7%) occur in the *HRAS* gene (mainly in the oral cavity and salivary gland tumors and associated with advanced stages of tumors), with the other two family members, *KRAS*, at 2.89% (mostly in syn-nasal tumors and often associated with HPV infection), and *NRAS*, at 2.20% (predominantly in nasopharyngeal tumors) (69). Members of the *RAF* gene family undergo fewer mutations (in about 3% of cases) compared to RAS genes, with the most known mutations reported in the *BRAF* gene (V599/600E, G468/469A, and Q257R) (58). In HNC, 6% of cases have heterozygous mutations in exons 12 and 13. Because KRAS activates only the wild-type *BRAF* gene, mutations in *KRAS* and *BRAF* genes never occur together in HNC and are redundant (58).

Many inhibitors targeting this pathway have been developed, essentially modifying the therapeutic strategy of cancers. Starting from *compound 12*, first reported by the Schokat group in 2013 (73), a series of inhibitors based on the *compound 12* structure are developed, such as *ARS-853* and *ARS-1620* (74, 75). The KRASG12C-specific drug, *AMG510 (storasib)*, first went into clinical trial in 2019 and was subsequently proven by the FDA in 2021 (76, 77) (**Table 1**).

**Table 1:** Clinical trials for HCN targeted therapies.

No.	Drug	Mechanism of action	Disease	Outcomes/Expected results	Patients enrolled	Year	Clinical trials	References
1	Storasib	KRAS^G12C^ inhibitor	Metastatic NSCLC	Potential effective first-line therapy for subgroups of NSCLC patients	42	2022	NCT04933695	77
2	Dacomitinib	EGFR inhibitor	(EGFR)-driven advanced solid tumors	Progression free survival, duration of response	104	2021	NCT04946968	–
3	Vandetanib	antineoplastic kinase inhibitor	Precancerous head and neck lesions	Effect of vandetanib compared to placebo on microvessel density	20	2012	NCT01414426	Awaiting for results
4	Panitumumab	human monoclonal antibody against EGFR	Unresected LA-HNSCC	Panitumumab cannot replace cisplatin in the unresected stage III-ivb HNSCC treatment	152	2012	NCT00547157	78
5	Panitumumab	human monoclonal antibody against EGFR	LA-HNSCC	Panitumumab did not durably improve quality of life swallowing as compared with standard with cisplatin	320	2017	NCT00820248	79
6	Gefitinib	tyrosine kinase inhibitor	Advanced-stage or recurrent HNC	Gefitinib had marginally better results in terms of overall response and safety as compared to methotrexate	200	2021	–	80
7	Erlotinib	EGFR inhibitor	Hnscc	Feasible and safe therapeutic option	35	2022	NCT: CTRI/2020/02/023378	81
8	Afatinib and pembrolizumab	irreversible EGFR tyrosine kinase inhibitor	Platinum-refractory, recurrent, or metastatic HNSCC	Afatinib may augment pembrolizumab therapy and improve the ORR in patients with HNSCC	29	2022	NCT03695510	82
9	Everolimus and carboplatin-paclitaxel	mTOR inhibitor	La t3–4/n0 3 hnscc	Safe, major tumor responses, impacting tumor microenvironment	49	2013	NCT01333085	83
10	Taselisib	PI3K pathway suppression	Metastatic solid tumors	Favorable safety profile and early signs of promising activity	34	2017	–	84
11	Alpelisib	PI3K pathway inhibitor	Hpv-associated hnscc	Safety and preliminary efficacy evaluation	9	2022	NCT03601507	Awaiting for results
12	Alpelisib	PI3K pathway inhibitor	Recurrent/metastatic HNSCC	Assesing early antitumor activity	40	2024	NCT04997902	Awaiting for results
13	Buparlisib	class I PI3K inhibitor	Recurrent/metastatic HNSCC	Significant promise as a treatment strategy	53	2014	NCT01527877	85
14	WNT974	WNT- porcupine enzyme inhibitor	Advanced solid tumors	WNT974 influence immune cell recruitment to tumours; enhance checkpoint inhibitor activity	94	2024	NCT01351103	86
15	Ruxolitinib	clinical JAK1/2 inhibitor	Operable HNSCC	Anti-cancer effects	16	2023	NCT03153982	87
16	Pembrolizumab combined with tacitinib/parsaclisib	Janus kinase 1 inhibitor	Advanced solid tumors	Modest clinical activity; little effect on T-cell infiltration in the tumor	159	2020	NCT02646748	88
17	Crenigacestat (LY3039478)	Notch inhibitor	Advanced or metastatic solid tumors	Poorly tolerated; lowered dosing and disappointing clinical activity	94	2020	NCT02784795	89
18	Cetuximab plus bevacizumab	EGFR and VEGF monoclonal antibodies	HNSCC	Significant reduction in tumor vascularization	48	2012	NCT00409565	90
19	Bevacizumab	VEGF monoclonal antibody	Recurrent or metastatic solid tumors	Improved the response rate and progression-free survival with increased toxicities	403	2025	NCT00588770	91
20	IK-930	Oral TEAD Inhibitor Targeting the Hippo Pathway	Advanced solid tumors	Evaluation of the safety, tolerability, pharmacokinetics, pharmacodynamics, and preliminary antitumor activity	198	2025	NCT05228015	–

Another KRASG12C-specific covalent inhibitor, *MRTX849 (adagrasib)*, developed by the Mirati group, also went into clinical trial in 2019 (92). Cetuximab is a chimeric mouse-human monoclonal IgG1 antibody against the extracellular domain of EGFR that can inhibit the functions of EGFR and induce cancer cell death via antibody-dependent NK cell-mediated cytotoxicity (93). In 2006, FDA approved the combination of cetuximab with radiotherapy for the treatment of locally advanced (LA) - HNSCC (94). In a study published in 2023, the conjugate of cetuximab with IRdye700DX, which is activated by illumination at 690 nm, has been used successfully on near infrared photoimmunotherapy to a patient with local recurrence of nasopharyngeal squamous cell carcinoma (95). Cetuximab, received full FDA approval for the treatment of patients with *K-RAS* wild-type, *EGFR*-mutant HNSCC following reports that its addition to radiation therapy results in significant improvements in disease control and overall survival (96, 97). Other inhibitors of EGFR/ERBB1 tested for HNC treatment are *dacomitinib* and *vandetanib*. When used with radiotherapy, dacomitinib reduces tumor volume in HNSCC, while vandetanib, and cisplatin radiosensitizes tumor cells. Combining vandetanib with radiotherapy is more efficient than other monotherapies or combination therapies (98, 99).

Several monoclonal antibodies of EGFR are still under intensive investigation, including *panitumumab*, *nimotuzumab*, and *zalutumumab*. According to the results from CONCERT-2 and HN.6 trials, panitumumab cannot replace cisplatin when combined with radiotherapy for LA-HNSCC (78, 79). In addition, combining panitumumab with the standard chemoradiotherapy strategy failed to provide any benefit (100). Adding nimotuzumab to radiotherapy with or without cisplatin provided long-term survival benefits for up to five years and improved the complete response rate in LA-HNSCC patients (101). In a phase 3 clinical trial involving 536 LA-HNSCC patients, nimotuzumab plus cisplatin and radiotherapy significantly improved the locoregional control rate without negatively impacting the quality of life (102). The promising results strongly supported the addition of nimotuzumab to LA-HNSCC patients who are treated with cisplatin and radiotherapy. Another monoclonal antibody, zalutumumab, extended the survival time from 8.4 to 9.9 weeks in recurrent or metastatic (R/M) HNSCC patients who had failed platinum-based chemotherapy (103). Meanwhile, moderate-to-severe skin rash during zalutumumab treatment was related to superior OS, independent of HPV infection and p16 status (104).

Some small molecular inhibitors of EGFR are also under investigation for the management of HNSCC, including selective inhibitors (e.g., gefitinib, erlotinib) (80, 81, 105, 106) and dual-target inhibitors (e.g., afatinib, lapatinib, and dacomitinib) (82, 107).

### 2.4 PIK3–AKT–mTOR signaling pathway

**Figure 2:**
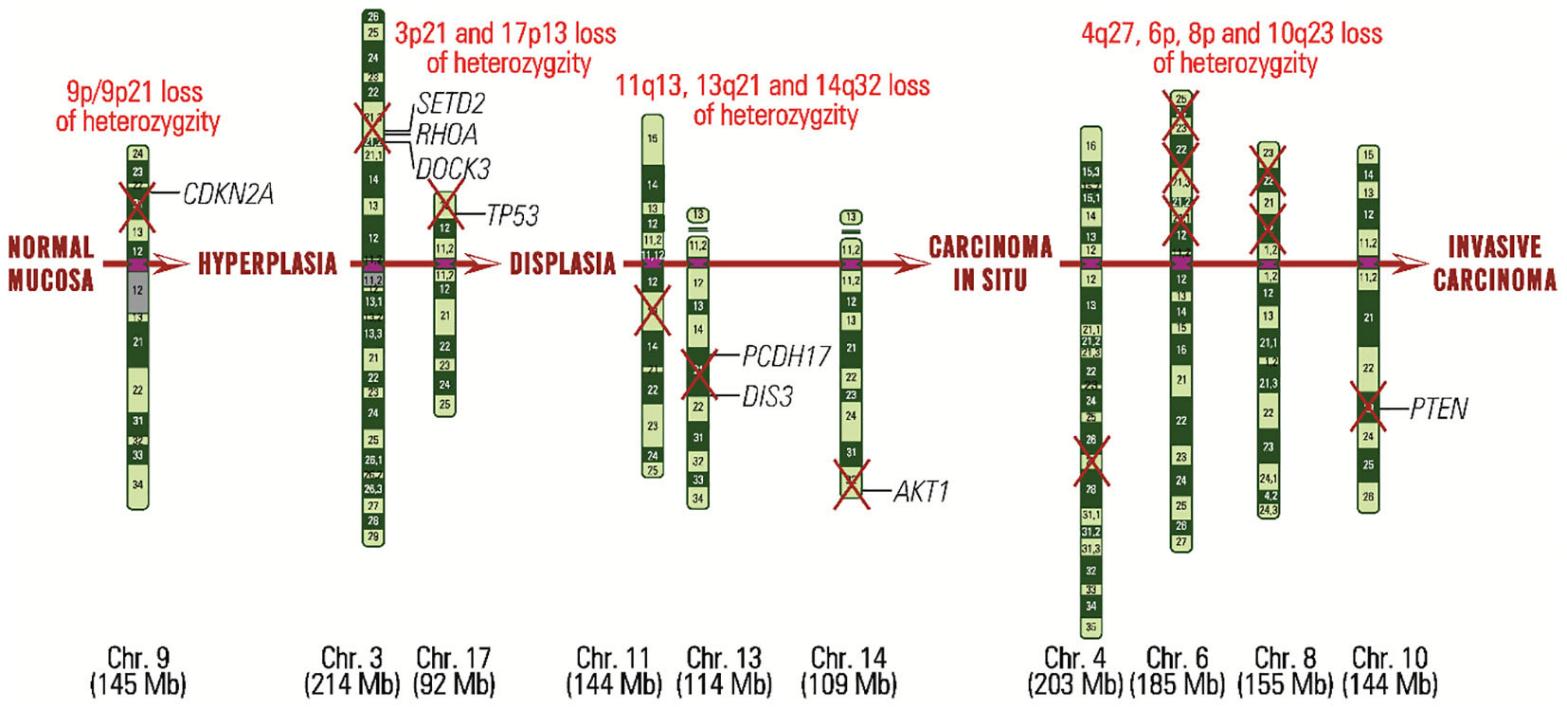
Carcinogenesis in HNC from normal mucinous stage to invasive carcinoma stage, highlighting each stage, chromosomal regions affected by loss of heterozygosity and genes that may be defective.

The *PIK3–AKT–mTOR signaling pathway* is very complex, being activated by extracellular signals represented by hormones, cytokines, and growth factors *via* receptors common with those of the RAS–RAF–MEK–ERK signaling pathway or *via* the enzyme PI3K (Phosphatidylinositol-4,5-Bisphosphate 3-Kinase), directly or indirectly, after PI3K activation by the GRB2, SOS, and RAS. This pathway is involved in cell growth, differentiation, survival, migration and proliferation, apoptosis evasion, and glucose metabolism (108). The central actor of this signaling pathway is the class I PI3K enzyme, which is part of the PI3K family of lipid and protein kinases, classified into three classes (I, II, and III). The class I enzymes function as secondary messengers in the intracellular transduction of biological signals and are more commonly associated with cancer (109, 110). The PI3K signals are transmitted through a phosphorylation cascade, represented by PIP2 (phosphatidylinositol-4,5-bisphosphate), which becomes PIP3 (phosphatidylinositol-3,4,5-trisphosphate), AKT/PKB (Serine/Threonine Kinase), and mTOR–RICTOR (Mechanistic Target Of Rapamycin Kinase–Rapamycin Insensitive Companion of mTOR) complex. Phosphorylation of AKT/PKB is antagonized by PTEN (phosphatase and tensin homolog) activity, which dephosphorylates the latter (111) and functions as an essential tumor suppressor (112). Subsequently, the phosphorylated AKT/PKB activates multiple downstream targets, promoting cell survival, activating anti-apoptotic pathways, and blocking apoptotic ones. PI3K and AKT can induce chromosome instability MET-dependently, an event suppressed by PI3K/mTOR inhibition, AKT depletion or PTEN overexpression (113). Interacting with HGF (Hepatocyte Growth Factor), MET can activate the PI3K–AKT–mTOR signaling pathway in endosomes independently of EGFR (114) or in the presence of TP53 mutants, as is mainly the case in patients with HPV-positive tumors (115). The *MET* gene is overexpressed in over 75% of HNSCC and has an increased copy number of 13%, associated with tumor progression and tumor dissemination in the early stages (116). Mutations of the *MET* gene are reported less frequently, but they seem to be involved in lymph node metastasis (117, 118). HNC may also carry somatic mutations in *PIK3R1* (~7%) and *PTEN* genes (51, 119), with loss of function in approximately 30% of HNC (more common than other cancers) due to mutations, loss of heterozygosity in the 10q region (which includes PTEN), detected in more than 70% of HNSCC, or hypermethylation, reported in ∼5% of these. In addition, TSC1/2 and LKB1 genes could be inactivated by loss of heterozygosity (*TSC1/2*), methylation (*TSC2*) and somatic mutations (*LKB1*) (119) (**Figure 2**).

Although Akt is recognized as a key player in cell migration and metastasis, its role remains controversial. Akt1 promotes cell migration in fibroblasts but inhibits it in breast cancer, while Akt2 has the opposite effects. The deletion of Akt isoforms can impact cancer progression differently depending on whether it is systematic or cell-autonomous (120, 121). Akt regulates cell migration through various mechanisms, such as phosphorylating PAK1, Girdin/APE, and ACAP1, which are involved in cytoskeletal dynamics and integrin trafficking, crucial for cell motility (122, 123). Further research is needed to fully understand these phosphorylation events and their implications in cancer metastasis.

Currently, drugs that target PI3K for *NOTCH1*-mutant tumors (124) or mTOR are undergoing clinical trials. *Everolimus* (RAD001), an allosterically inhibitor of only mTORC1 but not of mTORC2, is clinically used to treat various cancers, including previously treated recurrent or metastatic HNSCC (38, 83). *BKM120*, an oral, highly specific pan-Class I PI3K inhibitor, has strong antiproliferative characteristics in tumor cell lines (125). Previous studies with the PI3Kα inhibitors *taselisib*, *TAK-117*, and *alpelisib* in patients with solid tumors have reported promising clinical results (84, 126). *Taselisib* treatment showed an overall rate response (ORR) of 36% in patients with *PIK3CA* mutations but 0% in patients without *PIK3CA* mutations (84). The phase I trial with *alpelisib* specifically enrolled patients with *PIK3CA* mutations and showed an ORR of 6% and a stable disease rate of 52% (127, 128). Two phase II clinical trials, including to date 47 patients, are currently evaluating the clinical efficacy of alpelisib as monotherapy in HPV-positive HNSCC (NCT03601507) and alpelisib in combination with the farnesyltransferase inhibitor *tipifarnib* in *HRAS-* and *PIK3CA*-mutant HNSCC (NCT04997902; 129). *Buparlisib* (BKM120), another pan-PI3K inhibitor, exhibited limited antitumor activity in patients with HNSCC, with a disease control rate of 49% and an ORR of only 3% (NCT01527877; NCT01737450) (85, 130). Another phase II trial, including patients with HNSCC, revealed a modestly longer median in patients receiving a combination of buparlisib and paclitaxel than in the paclitaxel and placebo group (NCT01852292) (131). The buparlisib/paclitaxel combination is currently in phase III trial in patients with HNSCC (NCT04338399). Understanding the intricate interplay between Akt, Rho GTPases, and their downstream effectors, such as PAK1, as well as the regulatory networks controlling RhoA expression, is essential for uncovering novel therapeutic targets and strategies for combating metastatic cancer.

### 2.5 WNT/beta-catenin signaling pathway

The *WNT*(Wingless-Type)/*beta-catenin signaling pathway* is an alternative to the PIK3–AKT–mTOR pathway for promoting cell proliferation and avoiding apoptosis that includes three main pathways, the canonical WNT/b-catenin signaling pathway and the WNT/Ca2C and WNT/PCP non-canonical pathways (132–134). They also promote dysplastic transformation [WNT3, in oral leukoplakia (135), deterioration in histological grade, progression of clinical stage, and heightened metastatic potential in cervical lymph nodes [WNT3A, in laryngeal squamous cell carcinoma (136)], amplification of migration and invasiveness [WNT5A (137) and WNT5B (138), in OSCC], tumorigenesis and metastasis [WNT5A, in nasopharyngeal carcinoma (139) and laryngeal squamous cell carcinoma (137)], migration [WNT7A, in OSCC (140)], proliferation and invasiveness [WNT7B, in OSCC (141)], cell growth and survival and inhibition of apoptosis [WNT10B, in HNSCC (142)]. In some OSCC, WNT11 is involved in tumor suppression (143). Defects in the *WNT11* gene are found in 5% of OSCC, consisting of duplications, deep deletions or punctiform mutations affecting its tumor suppressor function. Other *WNT* genes are affected in smaller proportions (144).

Porcupine (Porc) is a membrane-bound *O*-acyltransferase (MBOAT) by whose Wnt ligands palmitoylation takes place, a process allowing them to be further secreted and recognized (145, 146). Therefore, repressing Porc can be a promising solution against tumors with aberrant Wnt/β-catenin activation (147). WNT974 is a potent, selective, and orally bioavailable first-in-class inhibitor of Porcupine with preclinical activity in Wnt-dependent HNC (86). Recently, Rodon and collaborators conducted a phase 1 study (NCT01351103) to investigate the safety and efficacy of the WNT974 in patients with solid tumors. The results of this clinical trial revealed that this inhibitor could be tolerated and may influence immune cell recruitment to tumors and enhance checkpoint inhibitor activity (86). In a phase I clinical trial, *OMP-18R5 (vantictumab)*, a monoclonal antibody targeting FZD receptors, inhibited tumor growth in HNC (148). In a phase Ib clinical trial, 54 patients with locally recurrent or metastatic HER2-negative breast cancer who were treated with weekly paclitaxel in combination with escalating doses of vantictumab were enrolled. The combination of vantictumab and paclitaxel was generally well tolerated and had promising efficacy. However, the incidence of fractures limits future clinical development of this particular WNT inhibitor in metastatic breast cancer (149). *XAV939*, a tankyrase inhibitor, inhibited β-catenin signaling attenuated cancer stem cells progression, consequently eliminating the chemical resistance in HNSCC (60, 150).

### 2.6 JAK–STAT signaling pathway

The *JAK–STAT signaling pathway* is involved in diverse physiological processes such as hematopoetic cell responses to cytokines (151), cell growth, proliferation and differentiation, survival, angiogenesis, and inflammatory or immune responses, but also in pathological conditions like tumor processes (152–154). The main proteins are JAKs (Janus kinases, named after the Roman god Janus, known to have two faces), with for members (JAK1, JAK2, JAK3 and TYK2, tyrosine kinase 2), and STAT (signal transducer and activator of transcription), with seven members (STAT1, STAT2, STAT3, STAT4, STAT5A, STAT5B, and STAT6) (155). The JAK–STAT signaling pathway is activated in many cancers, including HNC, mainly through overexpression of STAT1, STAT3, and STAT5, with the first correlating with favorable outcomes and the latter two with unfavorable outcomes (33, 156). In OSCC, overexpression of STAT3 and its accumulation in the nucleus is associated with reduced survival or favorable prognosis (157), promoting tumor angiogenesis by stabilizing and modulating the activity of HIF1 (hypoxia-inducible factor 1), which promotes the synthesis of VEGFs (158), a key protein involved in tumor invasiveness and metastasis. However, in breast cancers and some colorectal, spine, head, and neck cancers, activation of the JAK–STAT signaling pathway appears to result in a more favorable prognosis (159).

Several studies in human tumors and HNC cell lines have identified the JAK/STAT signaling pathway as a potential therapeutic target (160, 161). The group of Kowshik demonstrated that astaxanthin could hinder tumor progression by attenuating JAK/STAT signaling and its target molecules, including VEGF, cyclin D1, and MMP in the HPV-induced tumor models (162). The JAK1/2-selective inhibitor *ruxolitinib* is FDA-approved for several diseases, including myelofibrosis and graft versus host disease (163, 164). Currently, two multicenter, phase 1b clinical trials are undergoing to investigate the safety and efficacy of *ruxolitinib* (NCT03153982) and *pembrolizumab* (NCT02646748) in patients with HNC.

### 2.7 NOTCH signaling pathway

The *NOTCH signaling pathway* is activated by two families of ligands, Jagged (JAG1 and JAG2) and Delta-like (DLL1, DLL3 and DLL4). Upon interaction with NOTCH receptors (NOTCH1, NOTCH2, NOTCH3, and NOTCH4) they influence cell self-renewal capacity, cell cycle exit, survival, proliferation, and angiogenesis in a cell- and biological context-dependent manner (165–167). NOTCH1 signaling suppresses tumor development by promoting terminal keratinocyte differentiation and is probably protective in advanced stages of HPV-induced carcinogenesis, by reducing transcription of viral E6 and E7 genes (168). Conversely, NOTCH signaling causes FGF1-mediated tumor invasiveness in OSCC and increases mortality (167), probably through activation of MDM2, which ubiquitinates TP53 and primes it for degradation (169, 170). NOTCH function is negatively regulated by EGFR-activated C-JUN and inhibited by TP63, the latter being overexpressed in numerous cases of HNSCC (171). Notch signaling pathway is altered in 66% cases of HNSCC (172), *NOTCH1* gene presenting different percentages of mutations and genetics variants, some of which being nonsense and missense mutations (166, 173).

Several clinical trials were designed to target Notch signaling in patients with advanced solid tumors. An open-label phase 1a dose escalation clinical trial study (NCT01778439) of *brontictuzumab*, a monoclonal antibody, was designed to assess the safety, immunogenicity, pharmacokinetics, biomarkers, and efficacy of *brontictuzumab* in subjects with relapsed or refractory solid tumors. The group around Ferrarotto, who contributed to this clinical trial, reported significant clinical benefits in 6 of 36 patients, with four subjects having prolonged (≥ 6 months) disease stabilization. In addition, *brontictuzumab* was well tolerated at the maximum tolerated dose (174). More recently, two phase 1b studies with parallel dose-escalations (NCT02784795; NCT02836600) were designed to investigate the Notch inhibitor *crenigacestat* in patients with advanced or metastatic cancer from a variety of solid tumors. These clinical trials revealed that *crenigacestat* was poorly tolerated, leading to lowered dosing and limited clinical activity in patients with advanced or metastatic solid tumors (89, 175, 176).

### 2.8 Hypoxia and angiogenesis (HIF-VEGF) pathway

The *hypoxia and angiogenesis (HIF-VEGF) pathway* is essential for oxygen supply supplementation in solid tumors. The intense metabolic activities of solid tumors require an increased oxygen supply, which cannot be provided by the physiologically existing capillary structure of tissues. Thus, small tumors a few millimeters in diameter can be supplied by diffusion, but larger tumors with increased oxygen requirements rapidly enter hypoxia (177). In HNSCC, hypoxia is a common condition associated with poor prognosis and 5-year survival approaching 0% (178). Recently, Matic and collaborators searched potential biomarkers in HNSCC by examining mRNA expression of five highly upregulated (*CA9, CASP14, LOX, GLUT3, SERPINE1*) and four highly downregulated (*AREG, EREG, CCNB1*, and *KIF14*) hypoxia-responsive genes in 32 HNSCC tumors and six adjacent normal oral tissue. The results showed a significantly higher mRNA expression of the hypoxia marker *CA9* and *SERPINE1* in all tumor biopsies compared to normal tissue. Regarding the hypoxia-downregulated genes, the authors observed higher *KIF14* and *AREG* mRNA expression in HNSCC patients than in the the control group. In conclusion, the mRNA expression of *KIF14* could be a potential diagnostic marker and might serve as a predictor of treatment response in HNSCC (Matic et al., 2024). Signaling *via* the hypoxia and angiogenesis pathway begins with HIF1–2 proteins (hypoxia-inducible factors 1–2), which heterodimerize, and the HIF1–HIF2 heterodimers are translocated to the nucleus, where HIF1 promotes transcription of some genes, including the angiogenesis inductors VEGFA-D (Vascular Endothelial Growth Factors A-D) (67). Activation of HIF1 and HIF2 and overexpression of VEGFA-D in HNSCC result in carcinogenesis progression, increased aggressiveness, and poor prognosis, with a 2-fold increase of 2-year mortality risk (161).

*Bevacizumab*, a monoclonal antibody, is an FDA-approved VEGF inhibitor used in treating numerous cancers, either as a single agent or combined with chemotherapy or radiotherapy (179). In a phase III trial including 403 patients with HNSCC, adding bevacizumab to platinum-based chemotherapy significantly improved the response rate and progression-free survival. However, it did not increase the median survival rate (91). Several clinical trials have recently investigated the benefits of bevacizumab in combination with immune and chemotherapy. A phase II multicenter study (NCT03818061) aims to assess the effects of atezolizumab, a PD-L1 inhibitor, and bevacizumab in recurrent or metastatic HNSCC, considering the ORR. Another phase II trial (NCT00409565) compared bevacizumab with cetuximab, which has an immune-mediated activity. The results published by Argiris and collaborators revealed a significant reduction in tumor vascularization, with an ORR of 16%, a disease control rate of 73%, and a generally well-tolerated response (90). Three phase II trials are currently investigating the combination of bevacizumab, cetuximab, and chemoradiation in HNSCC (NCT00968435; NCT00703976; NCT01588431).

Tyrosine kinase inhibitors are small molecules acting by inhibiting several targets within angiogenic signaling pathways, including VEGFRs, EGFR, FGFR, and PDGFRs. In two phase II clinical trials, sorafenib and sunitinib, a multi-kinase inhibitor, were investigated in HNSCC patients and revealed minimal response rates (180, 181). A phase II trial of axitinib demonstrated a low objective response rate but a favorable disease control rate of 77% and median overall survival (OS) of 10.9 months with an acceptable toxicity profile (182, 183). Aurora kinase inhibitors are tested for RB-deficient, HPV-positive HNSCCs (184).

### 2.9 Hippo signaling pathway

Identified nearly three decades ago during tissue growth screening in *Drosophila melanogaster*, the Hippo signaling pathway is evolutionarily conserved in mammals (185, 186). Under physiological conditions, the Hippo signaling pathway restricts tissue growth in adult organisms by modulating cell proliferation, differentiation, and migration in growing organs (185). Its deregulation plays an important role in several diseases, including cancer and various organ-specific diseases (187, 188). In mammals, the pathway involves over 30 proteins, such as MST1/2, SAV1, MOB1A/B, and LATS1/2. These proteins phosphorylate YAP and TAZ, tagging them for cytoplasmic degradation and preventing their nuclear translocation, thus inhibiting transcription via TEADs and SMADs. Activation occurs through FAT1, KIBRA, AJUBA, NF2, RHO, AMPK, or by inactivation of STRIPAK complexes, which regulate MST1/2 and MAP4Ks (189, 190). In head and neck squamous cell carcinoma, common aberrations include mutations in FAT1, WWTR1/TAZ, YAP1, and MST2 (191, 192). Since 2016, several molecules targeting the Hippo pathway have been developed. In nasopharyngeal carcinoma, MGH-CP1, an inhibitor of TEAD2/4 auto-palmitoylation, is in preclinical trials, reducing TEAD4-mediated AKT signaling and inhibiting cell migration, invasion, and resistance to cisplatin (50, 189).

## 3 Epigenetic mechanisms

Acetylation is the transfer of an acetyl group from acetyl-CoA to the amino epsilon group of lysine residues by a histone acetyltransferase, neutralizing the positive charge of lysine residues and exposing DNA to transcriptional complexes (193). Other modifications consist of ubiquitination of lysine residues, phosphorylation of serine residues, SUMOylation of lysine residues (covalent interaction of a member of the SUMO, small ubiquitin-like modifier, protein family through an enzymatic cascade analogous but not similar to ubiquitination), and methylation of lysine and arginine residues (194, 195).

In HNSCC, global hypomethylation is more common in HPV-negative tumors. It is associated with genetic instability, including genome-wide loss-of-heterozygosity (LOH), single nucleotide polymorphisms (SNP), and alternative oncogenic pathways (196). Global hypomethylation was also linked with female gender and worse survival, predominantly for older patients with a stage I or II AJCC (American Joint Committee on Cancer) tongue squamous cell carcinoma without lymph node involvement and with postoperative radiotherapy (197). Assessment of the methylation status of *CALML5*, *DNAJC5G*, and *LY6D* genes identified in ctDNA from HNSCC patients demonstrated substantial predictive value in early cancer diagnosis (198). *FAM135B* (Family with Sequence Similarity 135 Member B) methylation appears to be associated with good prognosis, while *APBA1/MINT1* (Amyloid Beta Precursor Protein Binding Family A Member 1), *MINT31* (Methylated IN Tumors locus 31) *DCC* (Deleted In Colorectal Carcinoma Netrin 1 Receptor) methylation with poor prognosis, the latter one being also associated with bone invasiveness in the mandible (199). Binding KRAS and having a role in apoptosis induction, *RASSF2* (Ras association domain-containing protein 1) is intensely methylated. Other intensely methylated genes are *EDNRB* (Endothelin Receptor Type B), methylated in 97% of primary HNC tissues, and *RARB* (Retinoic Acid Receptor Beta), involved in transcriptional control (199, 200), and the tumor suppressor genes *PTEN*, *DAPK* (death-associated protein kinase), *MGMT* (O6-methylguanine-DNA methyltransferase), involved in DNA repair, *CDH1/ECAD* (E-cadherin), involved in cell adhesion, and *RASSF1* (Ras association domain-containing protein 1), involved in cell cycle control, apoptosis, and cell adhesion, inactivation of which is present in several cancers (201).

Normally, cells of HNSCC are hypoacetylated (e.g., H3K9ac in OSCC) (201) compared to normal mucosal cells, but histones in these cells can be acetylated by factors secreted by endothelial cells, in a paracrine manner. Consequently, acetylation induces the amplification of BMI1, a transcriptional repressor associated with poor survival and tumor aggressiveness, and vimentin, which marks the epithelial-mesenchymal transition (202). Deacetylation is mediated by histone deacetylases (HDACs), and HDACs inhibition *in vitro* results in fewer cancer stem cells (CSCs) in HNC. Thus, HDACs inhibition seems a promising strategy to disrupt the population of CSCs in HNC to create a homogeneous population of tumor cells characterized by well-defined biological traits and predictable behaviors (203). Several HDAC inhibitors are currently under evaluation in clinical trials for their efficiency in HNC treatment. When combined with chemoradiation therapy, vorinostat showed positive effects in HNC (NCT01064921). Additionally, abexinostat is under evaluation with pembrolizumab in an ongoing phase 1b dose-escalation trial for advanced solid tumors, including metastatic HNSCC (NCT03590054).

## 4 Non-coding RNAs

Non-coding RNAs do not encode proteins but have enzymatic, structural, or regulatory functions and can control gene expression. Depending on the number of nucleotides, they are short (microRNA, miRNA) or long (lncRNA, long ncRNA). miRNA are 21–23 nucleotides in size and bind partially complementary regions of the 3’ untranslated regions of several hundred mRNAs, potentially interfering with gene expression and cell differentiation, proliferation, and apoptosis. Although their aberrant expression can trigger the malignant process, miRNAs can be used as tumor suppressors due to their function in neoplastic development (194). In HNC, the expression of numerous miRNAs is associated with poor prognosis, decreased survival time, metastasis, and other tumor processes. Thus, miR34 and miR17–92 overexpression and miR137 underexpression are involved in apoptosis. miR210 overexpression and miR29 underexpression are associated with genetic instability, miR21 overexpression, and miR210 underexpression are involved in evasion of the immune response, miR26, and miR218 underexpression are associated with inflammation. miR26, and miR125b underexpression are involved in metabolism, overexpression of miR21 and miR155 and underexpression of miR29 and miR139 promote proliferation. Overexpression of miR26, miR125b, miR200b, miR96 and let-7d and underexpression of miR139, miR218, miR29 and miR200 are associated with metastasis. Overexpression of miR31, miR96, miR205 and miR96 and underexpression of miR210, miR125b and let-7d induce resistance to radiotherapy and chemotherapy (204). Polymorphisms in miR-146, miR-149, miR-196, and miR-499 may increase the risk of non-smokers infected with HPV, overexpression of miR-21, miR-181b, miR-184, and miR-345 is associated with malignant transformation, and overexpression of miR-21, miR-34c, 184 and miR-155 promotes proliferation and evasion of apoptosis (205), with miR-21 targeting the tumor suppressor genes PTEN (Phosphatase and Tensin Homolog) and PDCD4 (Programmed Cell Death 4) in some cancers (206).

Long ncRNAs are sequences of more than 200 nucleotides that carry methyl-guanosine ends, are often spliced or polyadenylated, and may be involved in chromatin remodeling. Being regulated by associated transcription factors, they control the transcription or can guide chromatin modification complexes to bind to specific loci, silencing or activating gene expression (194). For example, MIR31HG lncRNA appears to promote HIF1A and P21 expression, inducing proliferation and tumorigenesis, and LINC00460 lncRNA promotes the proliferation of HNSCC cells and epithelial-mesenchymal transition-mediated metastasis. On the other hand, SLC26A4-AS1 lncRNA interferes with cell invasiveness, migration, and metastasis, with a tumor suppressor role (207)

Besides their utility as biomarkers, ncRNAs are also very good therapeutic targets because they interact with numerous molecules when altering different cellular processes within the tumor microenvironment (208). The miRNA-based treatment relies on the premise that diseases disrupt the miRNA profiles which can be restored to normal (208).

Interestingly, the use of anti-miRNAs (miRNA sponges, miRNA masks, or miRNA antagonists) to deplete oncogenic miRNA as well as of miRNA mimetic molecules to simulate endogenous tumor suppressor miRNAs, showed promising results in limiting cancer cell growth in different HNC experimental models (208–211). Several ncRNA therapeutics have reached clinical trials in other malignancies. For instance, MRG-106 (cobomarsen), a miR-155 inhibitor, showed remarkable efficiency and tolerability in a phase I clinical trial (NCT02580552) involving 15 patients with cutaneous T-cell lymphoma (212). Therefore, although a nascent field of research, administering certain RNA-based formulations alone or in conjunction with systemic therapies seems to be a promising strategy for combating the burden of HNC.

## 5 Conclusions

The highly heterogeneous nature of HNC poses significant challenges in patient management due to cellular origin and anatomical site diversity, multiple etiological factors, and often late-stage diagnosis, which limits therapeutic options and affects survival and quality of life. Recent advancements in understanding the pathogenesis and drug resistance mechanisms have led to the development of various therapies.

Chemotherapy, chemoradiotherapy, targeted therapy, and immunotherapy show varied efficacy based on HNC stage, comorbidities, age, and previous treatments. New small molecule inhibitors, developed as monotherapies or in combination with other treatments, have shown promising results with moderate adverse effects by targeting specific gene expressions. These inhibitors have demonstrated fewer side effects compared to traditional therapies like chemotherapy and radiotherapy, enhancing patient tolerance. Notable drugs, such as the EGFR-directed monoclonal antibody cetuximab, pembrolizumab, and nivolumab, have achieved full FDA approval.

Despite these advancements, the complex interplay of multiple cell-signaling pathways limits therapeutic responses. Continuous clinical trials are necessary to confirm the effectiveness of these therapies in diverse patient groups and stages of HNC, and to identify suitable prognostic biomarkers for better therapeutic strategies. Comprehensive genomic sequencing studies have revealed numerous mutations in key signaling pathways, highlighting the potential of ncRNAs as biomarkers in HNC management.

Further improvements in treatment responses are needed, and the clinical translation of new inhibitors remains crucial. Combining these new agents with traditional treatments holds significant potential. Advances in molecular approaches are expected to enhance the success rate of targeted therapies, offering better evaluations of their efficacy and opening new research directions in personalized medicine for HNC diagnosis and treatment.

Ongoing studies aim to refine the use of novel compounds in therapeutic strategies, enabling precise identification of patients likely to benefit from these treatments, thus improving outcomes and innovating HNC treatment approaches.

## Author contributions

MC: Writing – original draft, Writing – review & editing. MCC: Conceptualization, Funding acquisition, Writing – original draft, Writing – review & editing. CB: Writing – review & editing. COV: Writing – original draft, Writing – review & editing. REC: Writing – original draft, Writing – review & editing. SB: Writing – review & editing. RG: Writing – review & editing. GB: Writing – review & editing.
